# Development of a Directly Visualized Recombinase Polymerase Amplification–SYBR Green I Method for the Rapid Detection of African Swine Fever Virus

**DOI:** 10.3389/fmicb.2020.602709

**Published:** 2020-12-22

**Authors:** Shuai Zhang, Aijun Sun, Bo Wan, Yongkun Du, Yanan Wu, Angke Zhang, Dawei Jiang, Pengchao Ji, Zhanyong Wei, Guoqing Zhuang, Gaiping Zhang

**Affiliations:** ^1^College of Veterinary Medicine, Henan Agricultural University, Zhengzhou, China; ^2^Key laboratory of Animal Immunology of the Ministry of Agriculture, Henan Provincial Key laboratory of Animal Immunology, Henan Academy of Agricultural Sciences, Zhengzhou, China

**Keywords:** African swine fever, African swine fever virus, recombinase polymerase amplification, SYBR green, surveillance

## Abstract

African swine fever (ASF) is a lethal disease in swine caused by etiologic African swine fever virus (ASFV). The global spread of ASFV has resulted in huge economic losses globally. In the absence of effective vaccines or drugs, pathogen surveillance has been the most important first-line intervention to prevent ASF outbreaks. Among numerous diagnostic methods, recombinase polymerase amplification (RPA)-based detection is capable of producing sensitive and specific results without relying on the use of expensive instruments. However, currently used gene-specific, probe-based RPA for ASFV detection is expensive and time-consuming. To improve the efficiency of ASFV surveillance, a novel directly visualized SYBR Green I-staining RPA (RPAS) method was developed to detect the ASFV genome. SYBR Green I was added to the amplified RPA products for direct visualization by the naked eye. The sensitivity and specificity of this method were confirmed using standard plasmid and inactivated field samples. This method was shown to be highly specific with a detection limit of 10^3^ copies/μl of ASFV in 15 min at 35°C without any cross-reactions with other important porcine viruses selected. In summary, this method enables direct sample visualization with reproducible results for ASFV detection and hence has the potential to be used as a robust tool for ASF prevention and control.

## Introduction

Belonging to phylum Nucleocytoviricota (NCLDV), class Pokkesviricetes, order Asfuvirales, and family Asfarviridae, African swine fever virus (ASFV) is the only member of genus *Asfivirus*. ASFV is a double-stranded DNA virus with a genome of approximately 190 kb in size that encodes more than 160 open reading frames (ORFs; Dixon et al., 2019). The structure of an extracytoplasmic virus particle comprises genetic material, nuclear shell, inner lipid membrane, icosahedral capsid, and outer lipid membrane with a diameter of around 250–260 nm (Wang et al., 2019). ASFV causes African swine fever (ASF), which is acute and frequently lethal in domestic pigs and wild boars, and is characterized by high fever, hemorrhage syndrome, and a mortality rate of up to 100%. Firstly reported in Kenya in 1921, ASF has spread to Europe in the 1950s and subsequently to North America in the 1960s (Sanchez-Vizcaino et al., 2012; European Food Safety et al., 2017). ASF was introduced into Caucasus and Russia in 2007 (Malogolovkin et al., 2012; Sanchez-Vizcaino et al., 2012). Since August 2018 when ASF was firstly reported in China, which has the largest pig consumption market in the world (Ge et al., 2018), there have been numerous ASF outbreaks within a short period of time in more than 30 Chinese provinces, causing a huge economic loss (Zhao et al., 2019; Teklue et al., 2020). To date, ASF has spread to more than 80 countries, threatening the pig industries worldwide (Dixon et al., 2019).

Although much efforts have been invested in scientific research, a vaccine or drug that is safe and effective is still unavailable. Strict bio-security interventions, including pathogen surveillance, are currently the main ASF control measures (Bellini et al., 2016). Early and rapid detection of ASFV infection in commercial swine populations is a critical strategy to prevent virus spreading and ASF outbreak. Molecular and serology approaches like polymerase chain reaction (PCR) and enzyme-linked immunosorbent assay (ELISA) are currently the most extensively used diagnostic methods recommended by the World Organization for Animal Health (OIE; Oura CA, 2019). Among various detection methods, recombinase polymerase amplification (RPA) has been extensively developed and used as a novel emerging pathogen detection tool (Piepenburg et al., 2006). The enzymes employed in the RPA process include a recombinase, a single-stranded DNA-binding protein, and a strand-displacing DNA polymerase. A RPA reaction usually takes 10–30 min long at 20–50°C depending on different targets. Both RPA-based PCR and real-time quantitative PCR (qPCR) reactions are highly sensitive and specific (Fan et al., 2020). However, all methods developed so far using RPA for ASFV detection are based on gene-specific probes, which are considerably more expensive especially in a high-throughput setting with field samples. SYBR Green I is a highly sensitive, asymmetrical cyanine dye that binds to the minor groove of nucleotide acids (Bruijns et al., 2016). Depending on the levels of DNA concentration, the SYBR Green I–DNA complex exhibits different colors at varying intensities visible to the naked eye. Due to its simple, rapid, and cost-effective properties, RPA coupled with SYBR Green I endpoint staining detection method has been used in cancer molecular diagnosis, meat product identification, and bacterial typing (Liu et al., 2016; Cao et al., 2018; Singpanomchai et al., 2019).

B646L (*p72*), a conserved gene that encodes the major capsid protein, has been the most widely used target for ASFV detection (Fernandez-Pinero et al., 2013; Wang A. et al., 2020; Wang Y. et al., 2020). The homology of nucleotide and amino acid sequences of *p72* have been found to be more than 95.5 and 97.8% in different ASFV strains, indicating that *p72* is highly conserved (Yu et al., 1996). Since *p72* has been usually used for ASFV genotype characterization, most commercial ASFV detection kits rely on *p72* targeting using fluorescent probe-based qPCR (Oura et al., 2013).

This study aims to improve the efficiency of ASFV surveillance. A novel directly visualized SYBR Green I-staining RPA (RPAS) method that is capable of giving good signals at a linear dynamic range was developed for ASFV detection using *p72* standard plasmid as a template. This method showed no cross-reactions with most major porcine viruses, with comparable specificity and sensitivity to five commercial ASFV probe-based kits. In addition, this method is more superior than the OIE-recommended PCR method for detecting residual amount of environmental ASFV contaminants. Most importantly, this method is cost-effective with a low turnaround time, which can significantly improve the efficiency of ASFV detection.

## Materials and Methods

### Viruses

The *p72* (MK333180.1) sequence information is based on the HLJ strain (Pig/HLJ/2018). Pseudorabies virus (PRV) strain (HN1201), Japanese encephalitis virus (JEV) live vaccine strain (SA14-14-2), and porcine epidemic diarrhea virus (PEDV) strain were provided by the Henan Academy of Agricultural Sciences (Zhengzhou, China), while porcine reproductive and respiratory syndrome virus (PRRSV) SD16 strain and porcine parvovirus (PPV) strain were derived from our laboratory.

### Reagents

Three different real-time fluorescent quantitative PCR kits of ASFV were respectively purchased from Laipusheng (catalog number 001, Luoyang, China), Zhongdao Biotechnology (catalog number ZD-R-D1087, Zhengzhou, China), and Mingrida Technology (catalog number 001, Beijing, China). Two different real-time PCR rapid detection kits of ASFV were respectively purchased from BioTeKe, catalog number PR7901 (Beijing, China) and BIOER Technology (catalog number BSL04MIA, Hangzhou, China). Twist Amp^TM^ Basic was purchased from Twist Amp (catalog number, TABAS03kit, Cambridge, United Kingdom). SYBR^TM^ Green I Nucleic Acid Gel Stain (catalog number S7567) was purchased from Invitrogen (Carlsbad, CA, United States). EasyPureHiPure Plasmid MiniPrepKit (catalog number EM121-01) and EasyPure^®^ PCR Purification Kit (catalog number EP101-01) were purchased from TransGen Biotech (Beijing, China). PrimeScriptTM RT Reagent Kit and gDNA Eraser Kit (catalog number RR047Q) were purchased from TAKARA (Shiga, Japan).

### Primers

The RPAS primer pair for *p72* was designed with a size of 30–36 bp, a GC content of 20%–70%, and a *T*_*m*_ value of 50–100°C. The maximum allowable single nucleotide repeat length was set as 5 and based on a conserved region in *p72* by sequence alignment of 76 ASFV isolates (Supplementary Table 1). The forward primer (5′-CCGATCACATTACCTATTATTAAAAACATTTCC-3′) and reverse primer (5′-GTGTCCCAACTAATATAAAATTCTCTTG CTCT-3′), designed to amplify 254 bp of *p72*, were synthesized by Sangon Biotech Co. (Shanghai, China). OIE-recommended ASF diagnostic PCR and qPCR primers were used as outlined in the Manual of Diagnostic Tests and Vaccines for Terrestrial Animals, 2019 (Oura CA, 2019). Specific primers for clinically important PRRSV, JEV, PPV, PRV, and PEDV (Supplementary Table 2) were designed as previously reported (Li et al., 2019).

### Generation of pUC57-*p72* Standard Plasmid

AFSV *p72* (MK333180.1) was synthesized (Sangon Biotech Co., Shanghai, China) based on the genomic sequence of ASFV HLJ strain (Pig/HLJ/2018). Synthesized *p72* was inserted into pUC57 to generate standard plasmid pUC57-*p72*, which was then amplified and extracted by EasyPureHiPure Plasmid MiniPrep Kit (TransGen Biotech, Beijing, China). The concentration and purity of plasmid DNA were determined (Genewiz Biotech Co., Suzhou, China) by Qubit 4 Fluorometer (Thermo Fisher Scientific, MA, United States).

For standard curve generation, pUC57-*p72* plasmid was serially diluted to 10^0^, 10^1^, 10^2^, 10^3^, 10^4^, or 10^5^ copies/μl. The conversion between plasmid copy numbers and mass was calculated using the following formula: *m* = (*n*)(1.096e–21g/bp), where *n* = DNA size (bp); *m* = mass.

### Optimization of RPA Assay for ASFV Detection

To optimize ASFV RPA assay, various reaction temperatures (20, 25, 30, 35, 40, 45, and 50°C), time (0, 5, 10, 15, 20, 25, and 30 min), primer final concentrations (0.24, 0.36, 0.48, and 0.6 μM), and magnesium acetate solution final concentrations (84, 112, and 140 mM) were tested according to the protocol recommended by the manufacturer. A typical RPA reaction mixture (50 μl) contains 29.5 μl of Twist Amp^®^ Rehydration buffer, 2.4 μl of forward primer (10 μM), 2.4 μl of reverse primer (10 μM), 2.5 μl of 280 mM magnesium acetate solution, and 13.2 μl of template and ddH_2_O. RPA reactions were performed in a regular water tank set at a desired temperature. Each RPA assay includes a no template control (NTC), a negative control, and a positive control with technical replicates.

At the end of reaction, 25 μl of the RPA product was purified by EasyPure^®^ PCR purification kit. The remaining 25 μl of the RPA product was directly detected by the naked eye under plenty of natural light or under white fluorescent lamp after adding 2 μl of 400 × SYBR Green I (Cao et al., 2018).

### Sensitivity, Specificity, and Cost Assessment of RPA Assay

Standard plasmid pUC57-*p72* was serially diluted and used as a template. Reproducibility was tested through three independent experiments with triplicate reactions in each batch. For sensitivity assessment and optimizing the volume of SYBR Green I, the reactions were carried out using diluted pUC57-*p72* at different concentrations (1–10^5^ copies/μl). Twenty-five microliters of the RPA product was directly observed under plenty of natural light or under white fluorescent lamp after adding 1, 2, 3, or 4 μl 400× SYBR Green I, respectively.

For specificity assessment, specific primers of ASFV were used to detect PRRSV, JEV, PEDV, PRV, and PPV. Viral nucleic acid was extracted by PureLink^TM^ Viral RNA/DNA Mini Kit (Invitrogen, United States). Extracted RNA was reverse transcribed into cDNA using High-Capacity cDNA Reverse Transcription Kit with RNase Inhibitor (Applied Biosystems, United States). As controls, virus-specific primers were used to identify different viruses by PCR.

For cost assessment of each method, the price of one test in commercial kits was calculated. The cost of a single RPAS test was calculated based on the cost of one typical RPA reaction with 2 μl of 400× SYBR Green I. The price of the OIE-approved PCR method was calculated based on the cost of one PCR reaction plus the cost of one run of agarose gel electrophoresis.

### Evaluation of RPA Assay Using Field Samples

A total of 39 field samples, including spleen and kidney specimens, sera, nasal swabs, anal swabs, and feces, were collected from pigs recovered from ASFV infection. Environment samples were collected from feeds and houseflies in the farm house. ASFV in all collected samples was inactivated before being subjected for DNA extraction using the PureLink^TM^ Viral RNA/DNA Mini Kit. The extracted DNA samples were used in RPA reactions set at 35°C for 15 min, following which 25 μl of the RPA product was directly observed under plenty of natural light or white fluorescent lamp after adding 2 μl of 400× SYBR Green I. Samples showing orange color and yellow green color are recognized as negative and positive samples, respectively. The OIE-approved method and commercial detection kits were used to test clinical samples according to the recommended procedures.

### Statistical Analysis

Multiple alignment of DNA sequences was analyzed by ClustalW (BioEdit, Manchester, United Kingdom). All statistical analyses were performed with GraphPad Prism 8.3.0 software (GraphPad Software Inc., San Diego, CA, United States). A value of *P* < 0.05 was considered to be statistically significant.

## Results

### Optimization of RPA Assay

**FIGURE 1:**
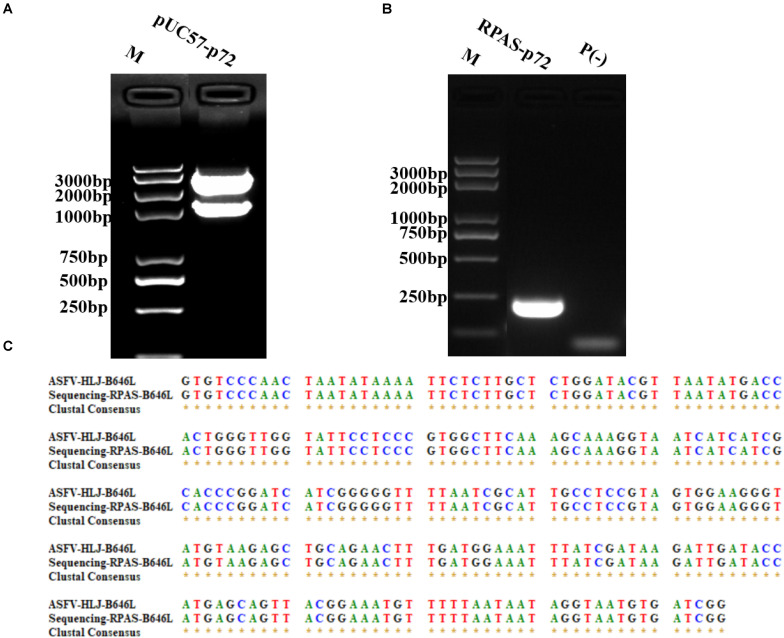
Construction and identification of pUC57-*p72*. **(A)** Dual restricted enzyme digestion analysis of pUC57-*p72*. **(B)** PCR amplification of *p72* using RPAS-specific primers. **(C)** DNA sequencing results of *p72*. M: DNA marker, P(-) = PCR control. These gel figures were modified for clarity and that the complete gels are available in Supplementary Figures 1,2.

The DNA construct of pUC57-*p72* was generated by inserting *p72* into pUC57 plasmid using restriction enzymes *EcoR*I and *BamH*I (Figure 1A). To identify the primers used in RPA reactions, pUC57-*p72* was used as the PCR template. The PCR results shown in Figure 1B indicated that the target fragment was successfully amplified by RPAS-specific *p72* primers and subsequently verified by DNA sequencing (Figure 1C).

**FIGURE 2:**
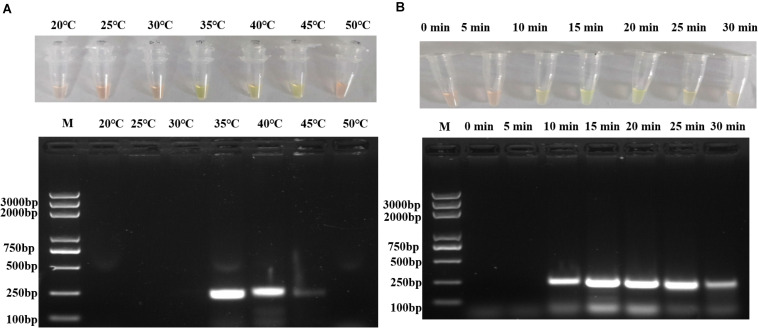
Optimization of RPA reaction temperature and time. **(A)** Different reaction temperatures in RPA assay and agarose gel electrophoresis. **(B)** Different reaction duration in RPA assay and agarose gel electrophoresis. Each figure is the representative of a triplicate in three separate experiments. M: DNA marker. These gel figures were modified for clarity and that the complete gels are available in Supplementary Figures 3,4.

To optimize the reaction temperature, seven different temperatures (20, 25, 30, 35, 40, 45, and 50°C) were evaluated in different reactions with 10^5^ copies/μl of pUC57-*p72* as the template. As shown in Figure 2A, the specific target fragment was successfully amplified at 35–45°C, with a higher amount of amplified product observed at 35–40°C than that at 45°C. Due to the ease and safety in operation, 35°C was chosen as the optimized reaction temperature.

**FIGURE 3:**
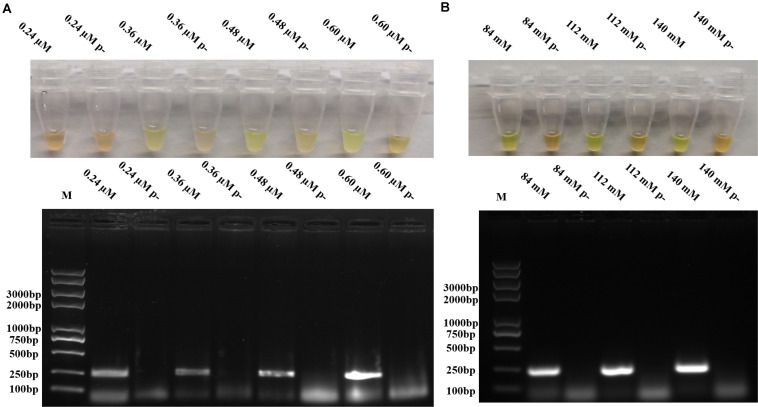
Optimization of RPA primer concentrations and magnesium acetate solution concentrations. **(A)** Different primer concentrations in RPAS assay and agarose gel electrophoresis. **(B)** Different magnesium acetate solution concentrations in RPAS assay and agarose gel electrophoresis. Each figure is the representative of a triplicate in three separate experiments. M: DNA marker. These gel figures were modified for clarity and that the complete gels are available in Supplementary Figures 5,6.

To optimize the reaction conditions, the assays were performed at different incubation times, primer concentrations, and magnesium acetate solution concentrations using 10^5^ copies/μl of pUC57-*p72* as the template. As shown in Figure 2B, RPAS assay with an incubation time of 10 min showed a positive signal, with the intensity of amplified DNA band density observed to be doubled in 15 min. It was found that a longer incubation time was incapable of increasing the signal intensity significantly and that a reaction time of 15, 20, 25, and 30 min, respectively, yielded a similar amount of RPA products. Since the amplification of target gene enlargement reached saturation in 15 min, the optimized reaction time for subsequent experiments was set at 15 min. As shown in Figure 3A, reactions with different primer concentrations could effectively amplify the target fragment. The amplification effect at a final primer concentration of 0.36 μM was comparable to that of 0.60 and 0.48 μM and superior to that of 0.24 μM; therefore, reactions with a final primer concentration of 0.36 μM were deemed to be more suitable. Under a final primer concentration of 0.36 μM, reactions with different magnesium acetate solution concentrations showed that the target fragment was successfully amplified at 84, 112, and 140 mM. However, the yield and specificity of the target product amplified with magnesium acetate solution at a concentration of 112 mM were better than those tested at different concentrations; hence, reactions with 112 mM magnesium acetate were determined to be relatively more suitable (Figure 3B).

Therefore, the RPA assay [containing 29.5 μl of TwistAmp^®^ Rehydration buffer, primer, 10 pmol/μl (1.8 μl, final concentration 0.36 μM), 5 μl of nucleic acid template, 10.1 μl of ddH_2_O, and 2 μl of 280 mM magnesium acetate solution (final concentration 112 mM)] was found to have an optimal reaction condition at 35°C for 15 min.

### Sensitivity and Specificity Evaluation of RPAS Assay

**FIGURE 4:**
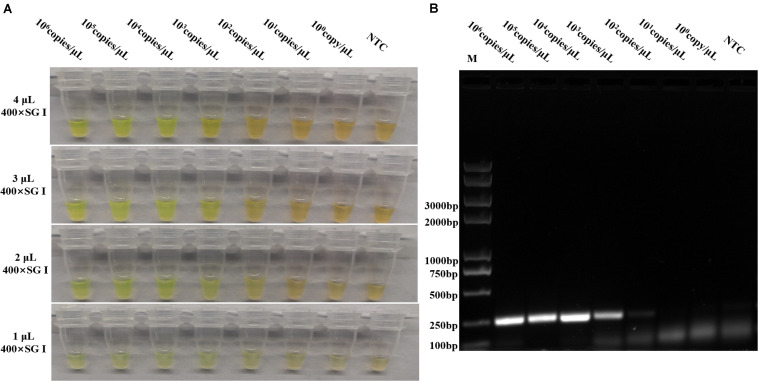
Sensitivity evaluation of the developed RPAS assay. **(A)** Sensitivity and SYBR Green I concentration evaluation using 10-fold serially diluted standard plasmid DNA solutions of pUC57-*p72* at concentrations ranging from 1 to 1 × 10^5^ copies/μl. **(B)** Agarose gel electrophoresis of RPA products in sensitivity evaluation. Each figure is the representative of a triplicate in three separate experiments. M: DNA marker. These gel figures were modified for clarity and that the complete gels are available in Supplementary Figure 7.

To optimize the volume of SYBR Green I and to evaluate the assay sensitivity and reproducibility, three independent biological repeats were conducted under the optimized conditions to detect *p72* using pUC57-*p72*, as well as pig tissue nucleic acids as the template in the reaction system. Different volumes of 400× SYBR Green I were individually added to 25 μl of RPA reaction products. As shown in Figure 4A, although 2–4 μl of 400× SYBR Green I was observed to yield positive staining, 2 μl was chosen because it was the lowest volume that yielded a clear change in color. As shown in Figures 4A,B, 10^3^ copies/μl of pUC57-*p72* were detected with stable and reliable amplification of *p72*, indicating that our optimized RPAS assay was sensitive and reproducible.

To further evaluate the specificity of this method, PEDV, JEV, PRRSV, PPV, and PRV were detected using the specific primers of each virus together with *p72* primers.

**FIGURE 5:**
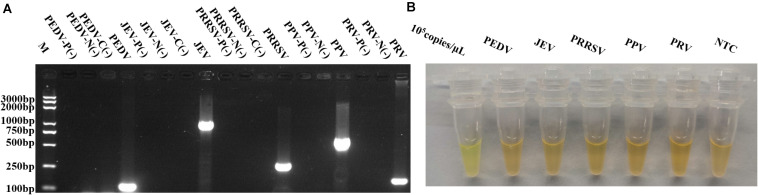
Specificity evaluation of RPAS assay developed. **(A)** Target DNA fragments from PEDV, JEV, PRRSV, PPV, and PRV were amplified using virus-specific primers. M: DNA marker, P(-) = PCR control, N(-) = nucleic acid extraction control, C(-) = reverse transcription control. **(B)** RPAS using *p72* primers. Each figure is the representative of a triplicate in three separate experiments. These gel figures were modified for clarity and that the complete gels are available in Supplementary Figure 8.

As shown in Figure 5A, while the target fragments of PEDV, JEV, PRRSV, PPV, and PRV were successfully amplified, the *p72* fragment was undetectable from these viruses, except ASFV, suggesting that the newly developed method was highly specific (Figure 5B).

### Sensitivity and Cost Comparison of RPAS Assay With Commercial Kits and the OIE-Approved PCR Method

To further evaluate the sensitivity of RPAS, ASFV ***p72*** was amplified by five different commercial qPCR kits and the OIE-approved PCR method using serially diluted standard plasmid DNA as the template. Our evaluation revealed that, while DNA template at a concentration of as low as 10^3^ copies/μl was detectable by both RPAS and the OIE-approved PCR, the minimum concentration of DNA template detected was 10–10^3^ copies/μl by the five probe-based commercial kits. These results indicated that RPAS had a similar level of sensitivity compared to that of commercial kits and the OIE-approved PCR method. With respect to cost, RPAS method is cheaper than other methods since it does not rely on large equipment (Table 1).

**TABLE 1:** Comparison of RPAS, OIE-recommended PCR, and commercial probe-based kits using pUC57-*p72* as the template.

Methods or kits	Sample							Cost/reaction ($)	Instrument requirement
	NTC	1.00E+00	1.00E+01	1.00E+02	1.00E+03	1.00E+04	1.00E+05		
RPAS method	–	–	–	–	+	+	+	3.27	Water bath
OIE–approved PCR method	–	–	–	–	+	+	+	1.40	PCR and electrophoresis system
A kit	–	–	+	+	+	ND	ND	5.53	Fluorescence quantitative PCR instrument
B kit	–	–	+	+	+	ND	ND	5.76	
C kit	–	–	–	+	+	ND	ND	5.67	
D kit	–	–	+	+	+	ND	ND	5.68	
E kit	–	–	–	+	+	ND	ND	4.20	

*“+” = positive, “–” = negative, and “ND” = not detected.*

### Evaluation of ASFV RPAS Assay Using Clinical Samples

A total of 39 field nucleic acid samples, including 26 extracted from tissues and sera, 9 extracted from excreta, and 4 extracted from environmental contaminants, were used as the assay template. As shown in Table 2, RPAS, OIE-recommended PCR, OIE-recommended qPCR, and commercial probe-based kit showed a positive rate of 74.36, 69.23, 79.49, and 71.79%, respectively (Table 2). The coincidence rate of RPAS with OIE-recommended PCR, OIE-recommended qPCR, and commercial probe-based kits was 92.31, 94.87, and 92.31%, respectively. Therefore, RPAS exhibited a level of sensitivity that is similar to that of OIE-recommended PCR and commercial probe-based kit.

**TABLE 2:** Comparison of RPAS, commercial probe-based kit, and OIE-approved PCR and qPCR using clinical samples.

Sample type	Result (positive/negative)			
	RPAS	OIE-PCR	OIE-qPCR	Kit
Animal tissue and serum	19/7	20/6	20/6	18/8
Animal excreta	8/1	6/3	9/0	8/1
Breeding environment	2/2	1/3	2/2	2/2
Total	29/10	27/12	31/8	28/11

## Discussion

Rapid and simple ASFV diagnostic method is critical for ASF outbreak control, especially in rural area. Probe-based qPCR method is currently widely used for ASFV diagnosis with high sensitivity and specificity. A number of isothermostatic amplification techniques, such as loop-mediated isothermal amplification assay (LAMP; Wang D. et al., 2020), cross-priming amplification (CPA; Wozniakowski et al., 2018), and cross-priming amplification combined with immunochromatographic strip method and polymerase cross-linking spiral reaction (PCLSR; Wozniakowski et al., 2017) visualized by SYBR Green dye, have been applied in ASFV nucleic acid detection. However, these methods are either expensive or complicated to be applied using field samples. In particular, requirements including high-cost reaction materials and instruments are unfavorable in low economic areas. RPA is a newly emerged isothermal molecular detection method that has been increasingly used, since it is rapid, simple, sensitive, specific, and importantly affordable to operate. Different methods can be used to distinguish RPA products. For example, RPA combined with lateral flow strip (RPA-LFD) diagnostic method has been reported in the detection of ASFV p72 and K205R genes. The RPA-LFD method is sensitive and specific without requiring the use of complicated instruments (Miao et al., 2019; Zhai et al., 2020). The use of gold nanoparticles, fluorescence-labeled probe, biotin, biotin-ligand, and antibodies has highly increased the cost in testing high-throughput clinical samples. Another drawback of using RPA-LFD assay for ASFV detection is the potential post-amplification contamination of samples in field settings (Miao et al., 2019).

To overcome the deficiencies outlined, we developed a rapid and simple ASFV detection method in the present study. Although previous primers used in RPA have shown high specificity and sensitivity, most of them are probe-based. Ideally, RPA primers should have a length of approximately 35 bases and a GC content of 30–70% without guanine or repeats at the 5′ terminus. We designed a pair of specific primer based on conserved sequences of p72 gene in 76 ASFV isolates as recommended by the manufacturer in the RPA system. The selected primers showed no unspecific reaction. In addition, other conditions including reaction temperature, duration, template amount, and primer concentration were also optimized. Amplified products under optimized conditions were compared using agarose gel electrophoresis to avoid subjective judgement. Our newly established method is very rapid and easy to operate by observation of color changes with the naked eye, while probe-based qPCR rely on expensive instruments. Our newly developed method is suitable to be applied in underdeveloped areas and countries where expensive instruments and other materials are not easily affordable. The limited sensitivity of RPAS assay with different detection methods may be improved with better dye for DNA staining in the future.

Our novel SYBR Green I-based RPA method for ASFV detection is significantly cheaper without the risk of potential sample contamination, while maintaining a high level of sensitivity and specificity (Table 1). Using the mixture of tissue-extracted genomic DNA and standard plasmid DNA as the template, our method has a detection sensitivity of up to 10^3^ copies/μl, which is better than that of OIE-approved regular PCR, and comparable to that of some commercial kits using clinical samples (Table 2). The potential defect of RPAS assay for ASFV is that SYBR Green I dye can bind to any double-stranded DNA, so the presence of template DNA in a high quantity may decrease the specificity of RPAS detection. To ensure the specificity of RPAS assay, previous studies have attempted to limit the quantity of DNA template in the reaction system (Liu et al., 2016). Meanwhile, a lower amount of PCR template may result in a high amount of PCR primer dimers that can bind SYBR Green I dye to produce potential false-positive results. However, we did not see any significant difference caused by primer dimers in our sensitivity and specificity evaluation (Figures 4, and 5). In addition, false positives may also occur under low concentration of SYBR Green I (Figure 4A), when the fluorescence signal is enhanced after SYBR Green I is combined with double-stranded DNA. An optimal amount of 400× SYBR Green I at 2–4 μl in a total reaction volume of 25 μl was used to avoid false positives caused by SYBR Green I. Conversely, false negatives may occur with low DNA content. The total amount of DNA template ranging from 300 ng to 2 μg in a 50-μl reaction system has been suggested to avoid false-negative and -positive results (Liu et al., 2016).

In summary, a novel RPA-based visible method for ASFV detection was developed and evaluated in this study. The detection capability of our method is better compared to that of the OIE-approved PCR method and comparable to those of commercial kits. Our highly sensitive and specific novel method is simple, rapid, and cheap to operate and therefore can potentially be applied in the diagnosis of ASFV.

## Data Availability Statement

The raw data supporting the conclusions of this article will be made available by the authors, without undue reservation.

## Ethics Statement

The animal study was reviewed and approved by the ethics committee of Henan Agricultural University. All samples were inactivated for genomic DNA extraction and detection.

## Author Contributions

SZ, GQZ, and GPZ designed the experiments and wrote and revised the manuscript. SZ, AS, BW, YD, YW, and AZ performed the experiments. SZ, AS, PJ, and ZW analyzed the data. BW and DJ provided samples. All authors contributed to the article and approved the submitted version.

## Conflict of Interest

The authors declare that the research was conducted in the absence of any commercial or financial relationships that could be construed as a potential conflict of interest.
