# Supplementary material for: Development of a Directly Visualized Recombinase Polymerase Amplification–SYBR Green I Method for the Rapid Detection of African Swine Fever Virus
Source: Front Microbiol. 2020 Dec 22;11:602709. doi: 10.3389/fmicb.2020.602709 (PMC7793706; doi:10.3389/fmicb.2020.602709)
Supplement: Supplementary file 1 [file Data_Sheet_1.pdf]

Supplementary Material

1 Supplementary Figures and Tables

1.1 Supplementary Table

Supplementary Table1 Multiple alignments among available ASFV p72 sequences of strain name and GeneBank accession number

GeneBank accession	ASFV isolate	GeneBank accession	ASFV isolate
KJ195685.1	Krasnodar 2012	NC_044946.	Ken06.Bus
AY578708.1	Za major	NC_044945.1	Ken05/Tk1
AY578707.1	wb	MN194591.1	ASFV/Kyiv/2016/131
AY578706.1	wart	MK128995.1	China/2018/AnhuiXCGQ
AY578705.1	vic	NC_044959.1	Georgia 2007/1
AY578704.1	ten	NC_044956.1	Benin 97/1
AY578703.1	Pr5	NC_044957.1	OURT 88/3(avirulent field isolate)
AY578702.1	Pr4	KX354450.1	47/Ss/2008
AY578701.1	o1	MK543947.1	Belgium/Etalle/wb/2018
AY578700.1	mk	NC_044955.1	47/Ss/2008
AY578699.1	M1	NC_044942.1	BA71
AY578698.1	kn	NC_044943.1	NHV
AY578697.1	ker	NC_044941.	L60
AY578696.1	K1	MK645909.1	ASFV-wbBS01
AY578695.1	ht	NC_001659.2	BA71V
AY578694.1	F6	LR722600.1	CzechRepublic 2017/1
AY578693.1	E75	LR722599.1	ASFV Moldova 2017/1
AY578692.1	E70	MN336500.1	RSA_2_2008

AY578691.1	cro3.5	MN394630.1	SPEC_57
AY578690.1	cro1.2	MK628478.1	ASFV/LT14/1490
AY578689.1	cam	KM102979.1	26544/OG10 from Italy
M34142.1 A	BA71V passed in Vero cell line CCL 81	MG939587.1	Pol17_03029_C201
KT795359.1	ETH/1a	MH910495.1	Georgia 2008/1
KT795358.1	ETH/2a	LS478113.1	Estonia 2014
KT795357.1	ETH/3a	MG939588.1	Pol17_04461_C210
KT795356.1	ETH/004	MH025919.1	N10
KT795355.1	ETH/017	MH025917.1	R7
KT795354.1	ETH/1	MH025916.1	R8
KT795353.1	ETH/AA	MH025918.1	R25
MK554698.1	VNUA HY-ASF1	MG939583.1	Pol16_20186_o7
MH713612.1	ASFV-SY18	MH025920.1	R35
L27499.1 A	Uganda	MK333180.1	Pig/HLJ/2018
L27498.1 A	Dominican Republic 1	LR536725.1	Belgium 2018/1
L76727.1 A	Dominican Republic 2	MK333181.1	DB/LN/2018

Supplementary Table 2 Primers of traditional PCR for PRRSV, JEV, PPV, PCV2, PRV and PEDV

Virus Primers	Sequence	Size of	References
PRRSV-275F	GGCCAGCCAGTCAATCAG	275bp	(Li et al., 2019)
PRRSV-275R	GGCAAACTAAACTCCACAGTG		
JEV-1044F	CAAACTGGCTCTGAAAGG		

JEV-1044R	TGTCTCAGGTCCATCTACG	1044bp
PPV-636F	ACATCTAAATATGCCAGAACACG	636bp
PPV-636R	GTTTGCCATGAGTGAGTTAATTT	
PRV-142F	CTCCTTGAGCGTCTTCGTCG	142bp
PRV-142R	CCTTCCTGTCCAACCCCTTC	
PEDV-106F	CGTTTTGCTGTCATTGTTCTT	106bp
PEDV-106R	AGACTAAACAAAGCCTGCCAATA	

1.2 Supplementary Figures

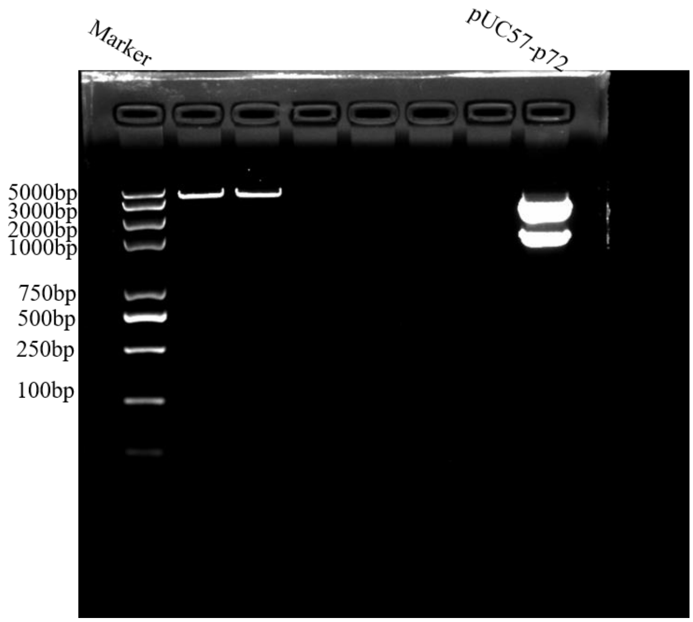

Supplementary Figure 1. (Original image) Dual restricted Enzymes digestion analysis of pUC57-p72 Plasmid. （M: DNA marker.）

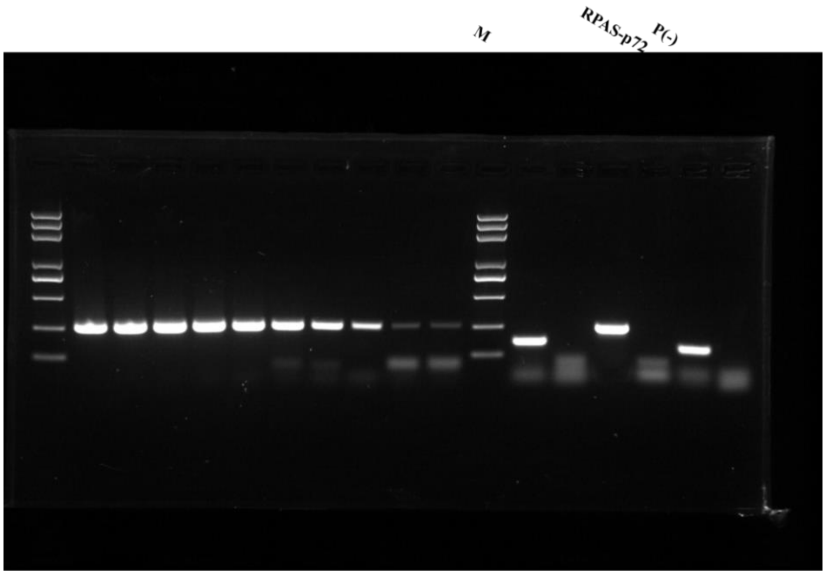

Supplementary Figure 2. (Original image) PCR amplification of p72 using RPAS specific primers.（M: DNA marker. P(-)=PCR control）

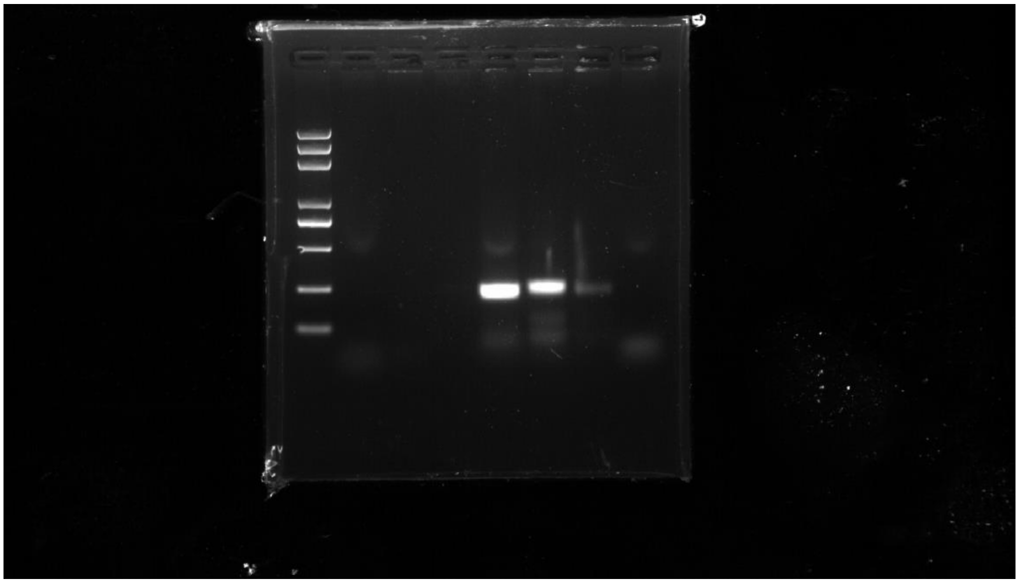

Supplementary Figure 3 (Original image) Different reaction temperatures in agarose gel electrophoresis assay.

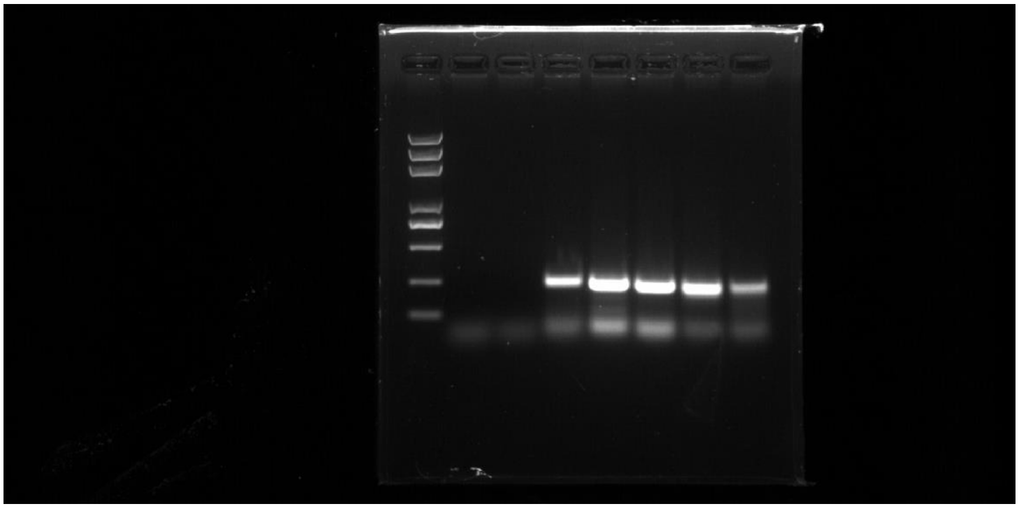

Supplementary Figure 4 (Original image) Different reaction duration in RPA assay and agarose gel electrophoresis assay.

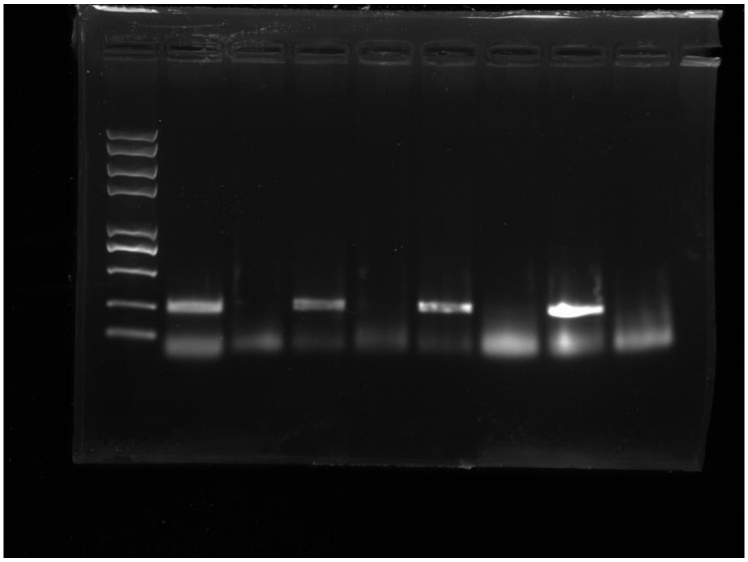

Supplementary Figure 5. (Original image) Different primer concentrations in agarose gel electrophoresis assay

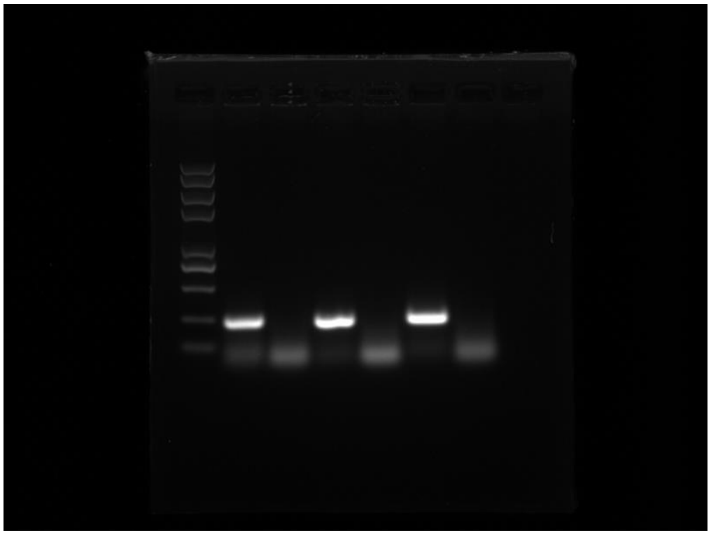

Supplementary Figure 6. (Original image) Different magnesium acetate solution concentrations in agarose gel electrophoresis assay.

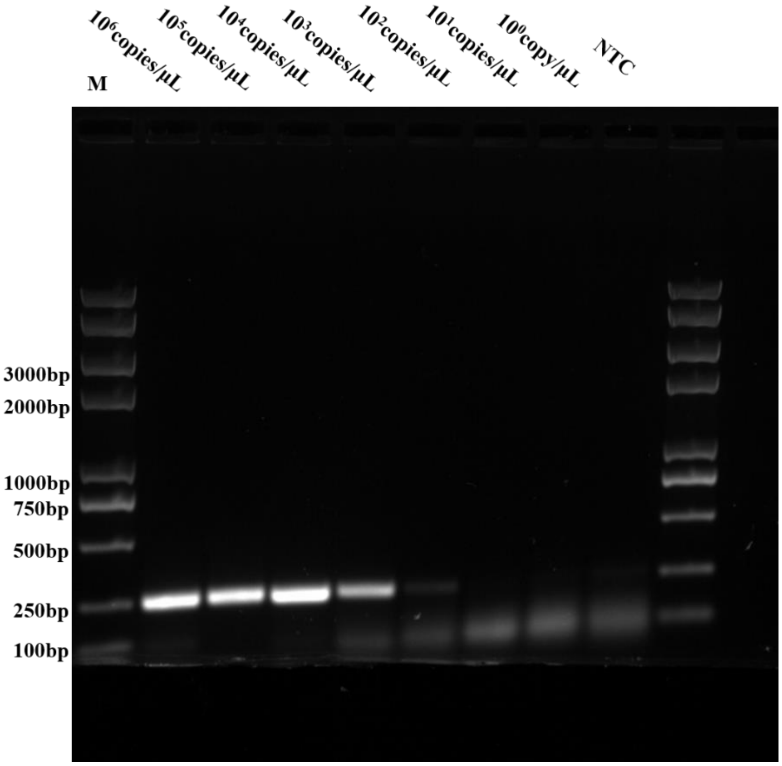

Supplementary Figure7 (Original image) Agarose gel electrophoresis of RPA Sensitivity, （M: DNA marker.）

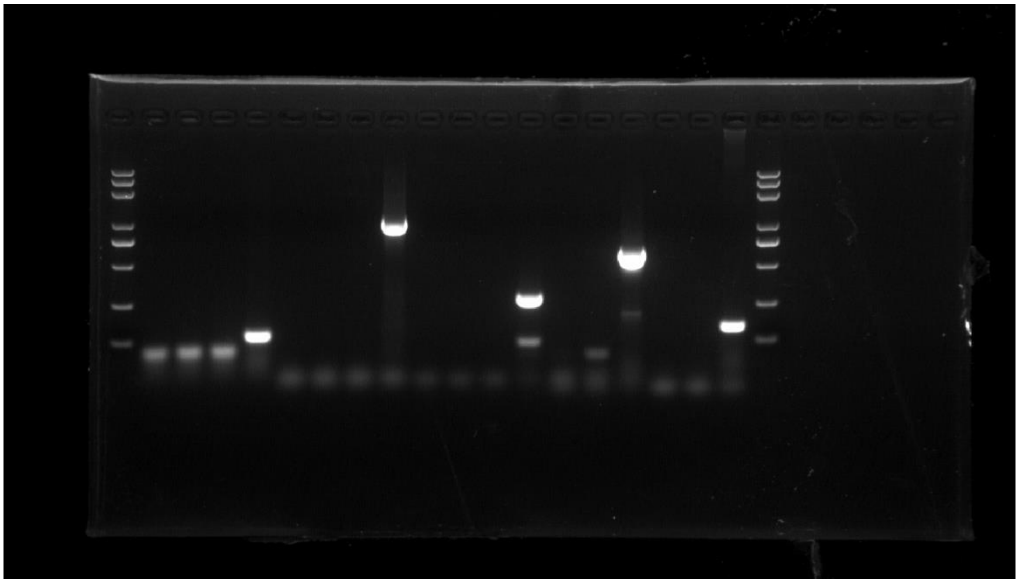

Supplementary Figure 8 (Original image) Target DNA fragments from PEDV, JEV, PRRSV, PPV and PRV were amplified using viral specific primers. M: DNA marker, P(-)= PCR control, N(-)= nucleic acid extraction control, C(-)= reverse transcription control.
